# Comprehensive Genetic Evaluation in Patients with Special Reference to Late-Onset Sensorineural Hearing Loss

**DOI:** 10.3390/genes15050571

**Published:** 2024-04-29

**Authors:** Ikuyo Miyanohara, Junichiro Ohori, Minako Tabuchi, Shin-ya Nishio, Masaru Yamashita, Shin-ichi Usami

**Affiliations:** 1 Department of Otolaryngology-Head and Neck Surgery, Kagoshima University Graduate School of Medical and Dental Sciences, 8-35-1, Sakuragaoka, Kagoshima 890-8520, Japan; k2304540@kadai.jp (J.O.); brown_mina_j@yahoo.co.jp (M.T.); yamashita@kufm.kagoshima-u.ac.jp (M.Y.); 2 Department of Hearing Implant Sciences, Shinshu University School of Medicine, 3-1-1 Asahi, Matsumoto 390-8621, Japan; nishio@shinshu-u.ac.jp

**Keywords:** non-syndromic hearing loss, next-generation sequencing, progressive hearing loss, diagnostic ratio, genetic hearing loss

## Abstract

Hearing loss (HL) is a common and multi-complex etiological deficit that can occur at any age and can be caused by genetic variants, aging, toxic drugs, noise, injury, viral infection, and other factors. Recently, a high incidence of genetic etiologies in congenital HL has been reported, and the usefulness of genetic testing has been widely accepted in congenital-onset or early-onset HL. In contrast, there have been few comprehensive reports on the relationship between late-onset HL and genetic causes. In this study, we performed next-generation sequencing analysis for 91 HL patients mainly consisting of late-onset HL patients. As a result, we identified 23 possibly disease-causing variants from 29 probands, affording a diagnostic rate for this study of 31.9%. The highest diagnostic rate was observed in the congenital/early-onset group (42.9%), followed by the juvenile/young adult-onset group (31.7%), and the middle-aged/aged-onset group (21.4%). The diagnostic ratio decreased with age; however, genetic etiologies were involved to a considerable degree even in late-onset HL. In particular, the responsible gene variants were found in 19 (55.9%) of 34 patients with a familial history and progressive HL. Therefore, this phenotype is considered to be a good candidate for genetic evaluation based on this diagnostic panel.

## 1. Introduction

Hearing loss (HL) is an etiologically heterogeneous disorder brought about by many causes such as genetic variants, aging, toxic drugs, noise, injury, and viral infections. HL is one of the most common deficits at birth, affecting approximately 1–2 in 1000 newborns [1]. Genetic factors are the most common cause for congenital- or early-onset HL, accounting for at least 60% of congenital sensorineural HL cases [1]. Among these genetic HL cases, the major form of inheritance is autosomal recessive, which is observed in about 75–80% of patients with non-syndromic sensorineural HL [2]. Several studies have focused on congenital- or early-onset HL patients for whom it is possible to achieve higher diagnostic yield [3,4,5,6,7]. In addition, some studies have focused on congenital- or early-onset severe-to-profound HL, for which it is generally possible to obtain a high genetic diagnostic rate [4,5,6,7]. In terms of the disease-causing mechanism, congenital severe-to-profound HL is well explained by genetic factors as pathogenic variants that occurred in the genes essential for the development of hearing function, leading to congenital severe-to-profound HL.

In contrast, the etiologies of late-onset and mild-to-moderate HL remain unclear. Unlike congenital- or early-onset HL, late-onset HL has been considered to result from a variety of factors including genetic and environmental factors. Late-onset HL occurs once hearing function has developed normally, with genetic or environmental factors damaging the maintenance of the hearing function, leading to the onset of HL. It was previously thought that the involvement of genetic factors is limited in late-onset HL. However, recent studies have indicated that a certain proportion of HL that presents after juvenile-onset HL or young adult-onset HL is also due to genetic causes [8,9,10,11].

Therefore, clarifying the genetic etiology, even in late-onset HL, has become more important than ever due to the clinical benefits in providing accurate diagnosis, prediction of HL severity, estimation of associated symptoms, appropriate treatment options, prevention of HL, and better genetic counseling [12]. Here, we report a consecutive prospective study based on target resequencing analysis for HL patients who visited our hospital between March 2007 and September 2022. Our study cohort mainly consists of juvenile- or young adult-onset mild-to-moderate HL patients. We believe our study results will be useful in furthering our understanding of the genetic background of HL patients in real practical clinical settings without any patient selection. In addition, our results are expected to shed some light on the etiology of juvenile- or young adult-onset mild-to-moderate HL.

## 2. Materials and Methods

### 2.1. Subjects

Ninety-four patients with HL who visited Kagoshima University hospital between March 2007 and September 2022 were enrolled in this study. We excluded three cases with other etiologies, so that 91 patients with HL and 106 relatives eventually participated in this study. Our university hospital is a tertiary referral hospital, and a wide range of patients in terms of age, particularly late-onset and post-lingual HL patients, are referred to our hospital for examination. In contrast, congenital- or early-onset HL patients identified as part of a newborn hearing screening program usually visit another medical center. Thus, our study cohort mainly consists of late-onset and post-lingual HL patients. This study was conducted with the approval of the ethics committees of Kagoshima University Graduate School of Medical and Dental Sciences and Shinshu University School of Medicine (Approval number: 718). Written informed consent was obtained from all patients (or from their next of kin, caretaker, or legal guardian in the cases of minors or children), and all procedures were performed in accordance with the Declaration of Helsinki Ethical Principles.

### 2.2. Clinical Evaluations

Clinical information was obtained retrospectively from medical records. Hearing thresholds were evaluated using pure-tone audiometry (PTA) and classified by pure-tone average over 500, 1000, 2000, and 4000 Hz. For infants or young children, conditioned orientation response audiometry and/or auditory brainstem response were performed. The severity of HL was classified into mild (20–40 dB HL), moderate (41–70 dB HL), severe (71–95 dB HL), and profound (>95 dB HL). The audiometric configurations were categorized into low-frequency, mid-frequency (U-shaped), high-frequency (gently sloping-type and steeply sloping-type), flat-type, and deaf, as reported previously [13]. With regard to age at onset, all patients were divided into 3 groups by age; the congenital/early-onset group (under 6 years old), the juvenile/young adult-onset group (6–39 years old), and the middle-aged/aged-onset group (over 39 years old).

### 2.3. Target Resequencing Analysis

DNA samples extracted from peripheral blood or saliva samples were used in this study. Next-generation DNA sequencing was performed for the 63 target genes [14] reported to be causative for non-syndromic hearing loss (Hereditary Hearing loss Homepage; http://hereditaryhearingloss.org/ accessed on 29 March 2024). We also analyzed 36 previously reported genes for syndromic HL if the patients presented with associated symptoms, as described previously [15]. The detailed protocols and DNA sequencing have been described elsewhere [14]. In brief, amplicon libraries were prepared using the Ion AmpliSeq Custom Panel, with the Ion AmpliSeq Library Kit 2.0 (ThermoFisher Scientific, Waltham, MA, USA) according to the manufacturer’s instructions. After amplicon library preparation, next-generation sequencing was performed with an Ion Proton or S5 sequencer (ThermoFisher Scientific) according to the manufacturer’s protocol. The sequence data were mapped against the reference human genome sequence (build GRCh37/hg19) with the Torrent Mapping Alignment Program (TMAP). The DNA variants were detected with the Torrent Variant Caller plug-in software (ThermoFisher Scientific).

After variant detection, annotation of identified variants was performed with ANNOVAR software [16]. The missense, nonsense, insertion, deletion, and splicing variants were selected from among the identified variants. Copy number variation analysis was also performed for all patients by using read depth data according to the copy number variation detection methods described in our previous report [17]. Variants were further selected as less than 1% of several control population databases including the 1000 genome database [18], the Genome Aggregation Database [19], the 1200 Japanese exome data in Human genetic variation database [20], the 38,000 Japanese genome variation database [21], and the 333 in-house Japanese normal hearing controls.

The pathogenicity of identified variants was analyzed in accordance with the American College of Medical Genetics (ACMG) standards and guidelines [22] with the ClinGen hearing loss clinical domain working group expert specification [23]. Variants were defined as candidate variants if the following criteria were fulfilled; (1) for the variants previously reported as “pathogenic” or “likely pathogenic” without any contradictory evidence, (2) novel variants classified as “pathogenic” or “likely pathogenic”, (3) variants of “uncertain significance” (VUS) identified as the only candidate after the filtering procedure without any candidate variants among the other 62 genes.

We performed Sanger sequencing analysis to validate the identified variants and confirm family segregations according to the manufacturer’s instructions. All PCR and sequencing primers were designed using the web version Primer 3 plus software [24].

## 3. Results

### 3.1. Patient Background and Identified Variants

The age at onset for this study cohort ranged from 0 to 64 years. In this study, we divided patients into three age groups (congenital/early-onset group, juvenile/young adult-onset group and middle-aged/aged-onset group). In general, HL onset under 6 years old is called “pre-lingual onset HL”, and this significantly affects language acquisition. We therefore categorized these patients into one group. It is difficult to differentiate the late-onset HL and presbycusis, so, we divided the other patients by the age of HL onset between 6 and 39 y.o. and over 39 y.o. The former group is thought to consist of late-onset HL patients without presbycusis cases, and the latter group is considered to consist of HL patients including presbycusis patients. The number of patients in each age group was as follows: 14 cases (15.4%) in the congenital/early-onset (onset at under 6 years old) group, 63 cases (69.2%) in the juvenile/young adult-onset (onset at 6–39 years old) group, and 14 cases (15.4%) in the middle-aged/aged-onset (onset at over 39 years old) group (Table 1). One of the unique characteristics of our study cohort was that 84.6% of the patients had late-onset HL.

There are 90 cases with sensorineural HL and one case with mixed HL, with 90 cases showing bilateral HL and one case presenting with unilateral HL. As for the severity, moderate HL was the most common, being observed in 41 cases (45.1%), followed by 32 cases with mild HL (35.2%). As for the audiometric configuration, flat-type HL was the most common, being observed in 32 cases (35.2%), followed by 21 cases with steeply sloping-type HL (23.1%), 20 cases with gently sloping-type HL (22.0%), 7 cases with U-shaped-type HL (7.7%), 5 cases with low-frequency type (5.5%), 3 cases diagnosed as deaf (3.3%), and 3 cases with different types of HL in the left and right ears (Table 1).

**Figure 1:**
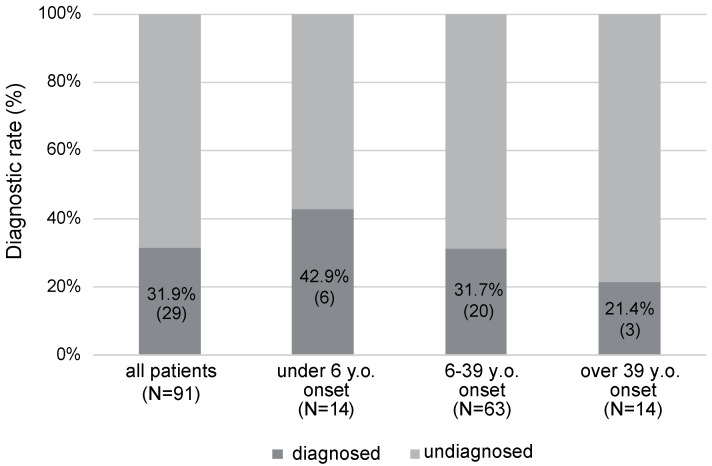
The diagnostic rates for each onset age group.

Based on the results of the next-generation sequencing analysis, we diagnosed 29 probands among the 91 participants, with the diagnostic rate for this study cohort being 31.9% (Figure 1). The diagnostic rate was highest in the congenital/early-onset group (42.9%), followed by the juvenile/young adult-onset group (31.7%), and the middle-aged/aged-onset group (21.4%) (Figure 1).

We identified 23 disease-causing candidate variants, with the most prevalent responsible genes identified in this study being a mitochondrial m.3243A>G variant and the *TMC1* gene, which were observed in 3 cases each, followed by 2 cases with *GJB2*, *CDH23*, *SLC26A4*, *STRC*, *MYO7A*, *ACTG1*, and *EYA4* gene variants, respectively, and 1 case each with *KCNQ4*, *MYO6*, *TECTA*, *USH2A*, *COCH*, *COL11A1*, *EYA1*, *NOG*, and *GRXCR1* variants (Table 2). Among the 23 identified variants, 21 variants had been reported previously, and 2 variants were novel.

### 3.2. Clinical Characteristics of Patients with Each Gene Variant

HL associated with the *GJB2*, *SLC26A4*, *STRC*, *TECTA*, *USH2A*, *NOG*, and *GRXCR1* genes was observed in patients with HL onset in the first decade. In contrast, all patients with *TMC1*, m.3243A>G, *ACTG1*, *KCNQ4*, *COCH*, *COL11A1*, *EYA1*, and *MYO7A* variants showed HL onset after the second decade. One case with an *ACTG1* variant and two cases with *MYO7A* variants became aware of their HL at over 40 years of age (Table 2).

Patients with *EYA1* variants showed mild hearing loss only in the high-frequency region, and these cases were categorized as normal hearing. HL associated with the *MYO6*, *TECTA,* and *COCH* genes was identified in patients with mild hearing loss. Patients with *STRC*, *MYO7A*, *KCNQ4*, *USH2*, *COL11A1*, and *NOG* gene variants showed moderate HL. In terms of audiometric configurations, *STRC*, *EYA4*, *TECTA*, *MYO6*, *USH2*, *GRXCR1*, *EYA1*, and *KCNQ4* gene variants were observed in patients with flat- or U-shaped-type HL. In contrast, patients with *TMC1*, *SLC26A4*, *COCH*, *ACTG1*, and *COL11A1* gene variants presented high-frequency impaired hearing loss, such as steeply sloping or gently sloping audiogram patterns (Table 2).

### 3.3. The Relationship between Diagnostic Rate and Phenotype

**Figure 2:**
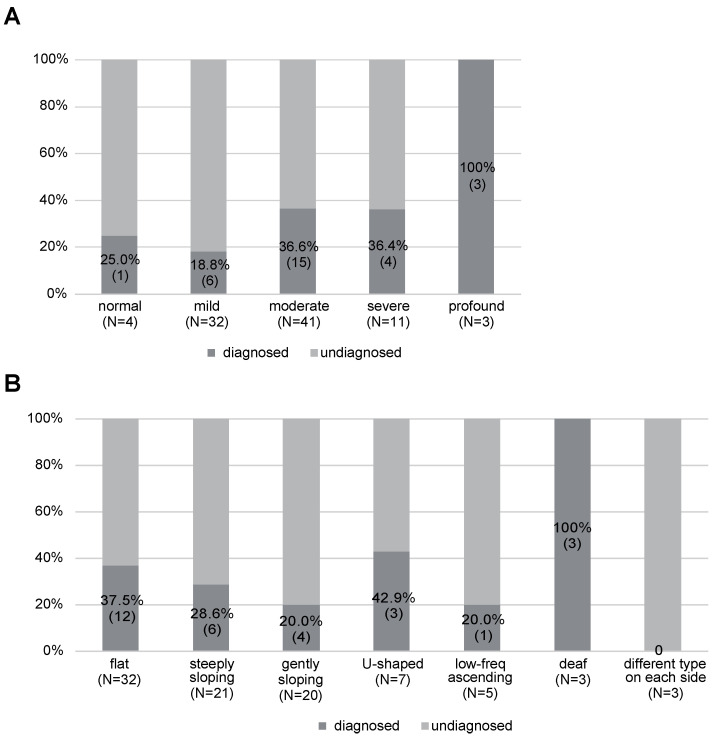
The diagnostic rates by severity and type of HL. (**A**) Diagnostic ratio for each severity of HL. (**B**) Diagnostic rate of for each audiometric configuration.

With regard to the diagnostic ratio for each HL severity, the profound HL group showed the highest ratio; i.e., we could identify the responsible gene variants for all three cases (100%), followed by the moderate HL (36.6%), severe HL (36.4%), normal (25.0%), and mild HL (18.8%) groups (Figure 2A). In terms of the diagnostic rate for each type of HL, the deaf-type showed the highest diagnostic ratio (100%), followed by U-shaped HL (42.9%), flat-type HL (37.5%), steeply sloping-type HL (28.6%), gently sloping-type HL (20.0%), and low-frequency ascending-type HL (20.0%) (Figure 2B). We could not identify any candidate variants for the patients with different types of HL on the right and left side.

**Figure 3:**
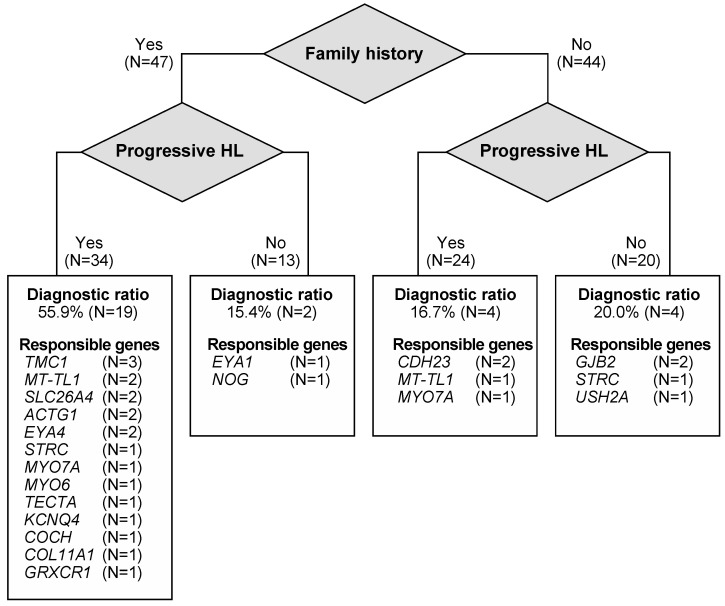
The diagnostic rate and responsible genes identified for each category of family history and progression of HL.

In addition to the severity of HL and type of HL, family history and progression of HL also affected the diagnostic ratio. There were 47 patients who have relatives with HL, and 44 patients did not have any affected family members. Among the 47 patients with a family history of HL, 34 patients had progressive HL. Similarly, among the 44 patients without a family history, 24 cases had progressive HL (Figure 3). The diagnostic rate was 55.9% for the 34 patients with a family history and progressive HL, which was significantly higher than that for the other groups (*p* < 0.01, Chi-square test) (Figure 3). Interestingly, most of the responsible genes identified in patients with a family history and progressive HL were autosomal dominant inheritance or maternal inheritance genes, demonstrating the importance of these genes in the patients included in this category.

## 4. Discussion

In this study, we analyzed 91 HL patients and identified 23 possibly disease-causing variants in 29 cases (31.9%). The diagnostic rate was the highest in the congenital/early-onset group (42.9%), followed by the juvenile/young adult-onset group (31.7%), and the middle-aged/aged-onset group (21.4%). A negative correlation was also observed between the age of onset (or awareness) and diagnostic yield in previous papers [8,9,10,11,45]. These findings emphasize that a genetic etiology is involved to a considerable degree even in patients with late-onset HL.

Previous etiological studies have shown that responsible genes such as *GJB2* and *SLC26A4* are most frequently identified [4,6]. This result reflects the composition of the study cohort with a particular focus on congenital- or pre-lingual-onset severe-to-profound HL. In this study, most of the participants suffered late-onset mild-to-moderate HL, and we are able to shed some light onto the genetic etiology of late-onset HL patients. The most prevalent responsible genes identified in this study were a mitochondrial m.3243A>G variant and the *TMC1* gene, which were observed in three cases each, followed by two cases with *GJB2*, *CDH23*, *SLC26A4*, *STRC*, *MYO7A*, *ACTG1*, and *EYA4* gene variants, respectively. The responsible genes identified in this study cohort were consistent with those in our previous report. Usami et al. reported that the frequent responsible genes for juvenile-onset HL were *KCNQ4*, Mitochondria m.3243A>G variant and m.1555A>G variant, *CHD23*, *MYO6*, *MYO7A*, *ACTG1*, *POU4F3*, and *WFS1* [9]. One of the major reasons for the large number of late-onset HL patients in this study is that genetic testing for HL has been covered by the social health insurance system in Japan since 2012. Thus, patients from a wide range of age groups and various levels of HL severity can consult specialists for genetic evaluation.

With regard to the relationship between the severity of HL and the diagnostic ratio, a positive correlation was observed. This result was consistent with that of a previous report [9]. In terms of the type of HL, the diagnostic rate was higher for U-shaped HL, flat-type HL, and deaf-type HL than for the other types, including high-frequency steeply sloping-type or gently sloping-type. It might be difficult to distinguish genetic HL from environmental HL in patients with high-frequency-associated HL. Age-related HL and cisplatin-induced HL generally lead to high-frequency HL. Thus, the high-frequency HL group will consist of patients other than those with genetic HL, including those with etiologies such as age, noise, and drugs as well as other environmental factors. Previously, the specific phenotype of a ski-slope audiogram patten, which is one type of high-frequency-associated HL, was also reported to result in a lower diagnostic rate [46]. In addition, both genetic and environmental factors may affect such HL. *CDH23* variants cause a wide range of phenotypes, from non-syndromic hearing loss (DFNB12) to syndromic hearing loss and Usher syndrome type ID (USH1D). In addition, several studies proposed that *CDH23* variants might modify the susceptibility to HL caused by the environmental factors such as age-related changes or noise exposure [47,48,49,50,51,52]. Thus, *CDH23* variants that are observed at high frequencies in the normal hearing control population may affect late-onset high-frequency HL in combination with environmental factors.

Interestingly, family history and progressive HL appear to be good markers for a higher diagnostic ratio. In this study, a higher diagnostic ratio was achieved in 19 (55.9%) of 34 patients with both a family history and progressive HL, even though most of these cases had late-onset mild-to-moderate HL. Among these 19 cases, 13 cases were diagnosed with autosomal dominant inheritance gene-associated HL, and 3 cases were diagnosed with maternal inheritance. In general, autosomal dominant non-syndromic HL is considered to be associated with post-lingual-onset, progressive HL [12], and our results were consistent with this hypothesis.

While useful findings were obtained, this study is limited by the small number of patients accumulated from the single institute. Further studies with a larger number of patients will be required to clarify the characteristics of each type of genetic HL.

## 5. Conclusions

In conclusion, we showed the utility of genetic testing even in the cases with late-onset HL. In particular, patients with late-onset mild-to-moderate HL with a family history and progressive HL appear to be good candidates for genetic testing. Our data will be useful in furthering our understanding of the genetic background of late-onset mild-to-moderate HL.

## Figures and Tables

**Table 1:** Patients’ characteristics in this study.

		Number	%
Sex	female	56	61.5
	male	35	38.5
Age at onset	under 6 y.o.	14	15.4
	6–39 y.o.	63	69.2
	over 39 y.o.	14	15.4
Family history of HL	yes	47	51.6
	no	44	48.4
Severity of HL	normal	4	4.4
	mild	32	35.2
	moderate	41	45.1
	severe	11	12.1
	profound	3	3.3
Audiometric configuration	flat	32	35.2
	steeply sloping	21	23.1
	gently sloping	20	22.0
	U-shaped	7	7.7
	low-freq. ascending	5	5.5
	deaf	3	3.3
	different type on each side	3	3.3

**Table 2:** Summary of the clinical characteristics and responsible gene variants identified in this study.

Family #	Relationship	Onset Age	Age	Inheritance	Gender	Severity of HL	Audiometric Configuration	Gene	RefSeq ID	Base Change	AA Change	References
1	proband	40	56	AD	F	Profound	Deaf	*ACTG1*	NM_001614	c.[791C>T];[=]	p.[P264L];[=]	Zhu et al., 2003 [25]
2	proband	12	60	AD	M	Moderate	Gently sloping	*ACTG1*	NM_001614	c.[952A>G;977A>G];[=]	p.[T318A;K326R];[=]	This study
	daughter	12	25	AD	F	Mild	Gently sloping	*ACTG1*	NM_001614	c.[952A>G;977A>G];[=]	p.[T318A;K326R];[=]	This study
3	proband	6	33	Sporadic	F	Moderate	Flat	*CDH23*	NM_022124	c.[4762C>T];[8866C>A]	p.[R1588W];[R2956S]	Miyagawa et al., 2012 [26], Usami et al., 2022 [27]
4	proband	15	35	Sporadic	F	Mild	Steeply sloping	*CDH23*	NM_022124	c.[4853C>T];[4853C>T]	p.[T1618M];[T1618M]	Usami et al., 2022 [27]
5	proband	12	31	AD	M	Mild	Steeply sloping	*COCH*	NM_004086	c.[1096G>A];[=]	p.[V366M];[=]	Usami & Nishio 2022 [9]
6	proband	15	41	AD	M	Moderate	Gently sloping	*COL11A1*	NM_001854	c.[3482dupC];[=]	p.[G1162Wfs*6];[=]	This study
	mother	50	65	AD	F	Moderate	Flat	*COL11A1*	NM_001854	c.[3482dupC];[=]	p.[G1162Wfs*6];[=]	This study
7	proband	27	27	AD	M	Normal	Flat	*EYA1*	NM_172058	c.[1081C>T];[=]	p.[R361X];[=]	Spruijt et al., 2006 [28]
	mother	2~3	57	AD	F	Profound	Deaf	*EYA1*	NM_172058	c.[1081C>T];[=]	p.[R361X];[=]	Spruijt et al., 2006 [28]
8	proband	10	38	AD	F	Severe	Flat	*EYA4*		1 copy loss		Shinagawa et al., 2020 [29]
	mother	26	66	AD	F	Severe	Flat	*EYA4*		1 copy loss		Shinagawa et al., 2020 [29]
9	proband	6	35	AD	F	Moderate	Flat	*EYA4*		1 copy loss		Shinagawa et al., 2020 [29]
10	proband	0	0	Sporadic	F	Severe	Flat	*GJB2*	NM_004004	c.[176_191del];[235del]	p.[G59Afs*18];[L79Cfs*3]	Abe et al., 2000 [30], Fuse et al., 1999 [31]
11	proband	7	8	Sporadic	F	Moderate	Gently sloping	*GJB2*	NM_004004	c.[235del];[235del]	p.[L79Cfs*3];[L79Cfs*3]	Fuse et al., 1999 [31]
12	proband	3	41	Sporadic	M	Severe	Flat	*GRXCR1*	NM_001080476	c.[439C>T];[784C>T]	p.[R147C];[R262X]	Mori et al., 2015 [32]
13	proband	12	33	AD	F	Moderate	U-shaped	*KCNQ4*	NM_004700	c.[909C>G];[=]	p.[F303L];[=]	Usami & Nishio 2022 [9]
	father	50	66	AD	M	Moderate	Steeply sloping	*KCNQ4*	NM_004700	c.[909C>G];[=]	p.[F303L];[=]	Usami & Nishio 2022 [9]
14	proband	30	33	Maternal	F	Moderate	Flat	*MT-TL1*		m.3243A>G		Goto et al., 1990 [33]
	daughter	Unaware	13	Maternal	F	Normal	Flat	*MT-TL1*		m.3243A>G		Goto et al., 1990 [33]
15	proband	22	32	Maternal	F	Moderate	Flat	*MT-TL1*		m.3243A>G		Goto et al., 1990 [33]
16	proband	18	40	Maternal	M	Profound	Deaf	*MT-TL1*		m.3243A>G		Goto et al., 1990 [33]
	mother	Unaware	65	Maternal	F	Moderate	Gently sloping	*MT-TL1*		m.3243A>G		Goto et al., 1990 [33]
17	proband	13	13	AD	M	Mild	U-shaped	*MYO6*	NM_004999	c.[2545C>T];[=]	p.[R849X];[=]	Sanggaard et al., 2008 [34]
	brother	9	9	AD	M	Mild	Flat	*MYO6*	NM_004999	c.[2545C>T];[=]	p.[R849X];[=]	Sanggaard et al., 2008 [34]
18	proband	45	68	AD	M	Moderate	Steeply sloping	*MYO7A*	NM_000260	c.[4118G>A];[=]	p.[R1373Q];[=]	Usami & Nishio 2022 [9]
19	proband	45	57	AR	F	Moderate	Flat	*MYO7A*	NM_000260	c.[359G>A];[874C>T]	p.[R120H];[R292W]	Oza et al., 2018 [23], Nykamp et al., 2017 [35]
20	proband	6	46	AD	F	Moderate	Low-freq. ascending	*NOG*	NM_005450	c.[645C>A];[=]	p.[C215X];[=]	Usami et al., 2012 [36]
21	proband	3	70	AR	F	Severe	Steeply sloping	*SLC26A4*	NM_000441	c.[107_116delinsTCGCTTT]; [1315G>A]	p.[H36_R39delinsLAPF]; [G439R]	Usami & Nishio 2022 [9], Suzuki et al., 2017 [37]
22	proband	3	65	Sporadic	F	Profound	Deaf	*SLC26A4*	NM_000441	c.[918+1G>A];[2162C>T]	p.[spl.];[T721M]	Van Hauwe et al., 1998 [38], Usami et al., 1999 [39]
23	proband	6	7	Sporadic	M	Moderate	Flat	*STRC*		2 copy loss		Yokota et al., 2019 [40]
24	proband	5	16	Sporadic	F	Moderate	Flat	*STRC*		2 copy loss		Yokota et al., 2019 [40]
25	proband	8	25	AD	M	Mild	U-shaped	*TECTA*	NM_005422	c.[5597C>T];[=]	p.[T1866M];[=]	Sagong et al., 2010 [41]
	daughter	NHS	4	AD	F	Mild	Flat	*TECTA*	NM_005422	c.[5597C>T];[=]	p.[T1866M];[=]	Sagong et al., 2010 [41]
	son	NHS	7	AD	M	Moderate	U-shaped	*TECTA*	NM_005422	c.[5597C>T];[=]	p.[T1866M];[=]	Sagong et al., 2010 [41]
26	proband	22	22	AD	F	Mild	Steeply sloping	*TMC1*	NM_138691	c.[1627G>A];[=]	p.[D543N];[=]	Moteki et al., 2016 [42]
	mother	20s	48	AD	F	Profound	Deaf	*TMC1*	NM_138691	c.[1627G>A];[=]	p.[D543N];[=]	Moteki et al., 2016 [42]
	grandfather	40s	77	AD	M	Profound	Deaf	*TMC1*	NM_138691	c.[1627G>A];[=]	p.[D543N];[=]	Moteki et al., 2016 [42]
	aunt	30s	46	AD	F	Severe	Steeply sloping	*TMC1*	NM_138691	c.[1627G>A];[=]	p.[D543N];[=]	Moteki et al., 2016 [42]
27	proband	18	21	AD	M	Mild	Gently sloping	*TMC1*	NM_138691	c.[1627G>A];[=]	p.[D543N];[=]	Moteki et al., 2016 [42]
	father	20s	48	AD	M	Severe	Gently sloping	*TMC1*	NM_138691	c.[1627G>A];[=]	p.[D543N];[=]	Moteki et al., 2016 [42]
28	proband	14	16	AD	F	Moderate	Steeply sloping	*TMC1*	NM_138691	c.[1627G>A];[=]	p.[D543N];[=]	Moteki et al., 2016 [42]
	father	14	45	AD	M	Profound	Deaf	*TMC1*	NM_138691	c.[1627G>A];[=]	p.[D543N];[=]	Moteki et al., 2016 [42]
29	proband	3	16	Sporadic	F	Moderate	Flat	*USH2A*	NM_206933	c.[5329C>T];[11389+3A>T]	p.[R1777W];[spl.]	Nakanishi et al., 2011 [43], Soens et al., 2017 [44]

NHS: newborn hearing screening, AD: autosomal dominant, AR: autosomal recessive, M: male, F: female, spl.: splicing.

## Data Availability

The datasets used during the current study are available from the corresponding author on reasonable request.
